# Impact of Stakeholders Influence, Geographic Level and Risk Perception on Strategic Decisions in Simulated Foot and Mouth Disease Epizootics in France

**DOI:** 10.1371/journal.pone.0086323

**Published:** 2014-01-21

**Authors:** Maud Marsot, Séverine Rautureau, Barbara Dufour, Benoit Durand

**Affiliations:** 1 Université Paris-Est, French Agency for Food, Environmental and Occupational Health and Safety (Anses), Laboratoire de Santé Animale, Epidemiology Unit, Maisons-Alfort, France; 2 Animal Health Unit, French General Directorate for Food (DGAL), Ministry of Agriculture, Food, Fisheries and Rural Affairs, Paris, France; 3 EPIMAI, Alfort National Veterinary School (ENVA), USC Anses, Maisons-Alfort, France; The University of Melbourne, United States of America

## Abstract

Comparison of control strategies against animal infectious diseases allows determining optimal strategies according to their epidemiological and/or economic impacts. However, in real life, the choice of a control strategy does not always obey a pure economic or epidemiological rationality. The objective of this study was to analyze the choice of a foot and mouth disease (FMD) control strategy as a decision-making process in which the decision-maker is influenced by several stakeholders (government, agro-food industries, public opinion). For each of these, an indicator of epizootic impact was quantified to compare seven control strategies. We then determined how, in France, the optimal control strategy varied according to the relative weights of stakeholders and to the perception of risk by the decision-maker (risk-neutral/risk-averse). When the scope of decision was national, whatever their perception of risk and the stakeholders' weights, decision-makers chose a strategy based on vaccination. This consensus concealed marked differences between regions, which were connected with the regional breeding characteristics. Vaccination-based strategies were predominant in regions with dense cattle and swine populations, and in regions with a dense population of small ruminants, combined with a medium density of cattle and swine. These differences between regions suggested that control strategies could be usefully adapted to local breeding conditions. We then analyzed the feasibility of adaptive decision-making processes depending on the date and place where the epizootic starts, or on the evolution of the epizootic over time. The initial conditions always explained at least half of the variance of impacts, the remaining variance being attributed to the variability of epizootics evolution. However, the first weeks of this evolution explained a large part of the impacts variability. Although the predictive value of the initial conditions for determining the optimal strategy was weak, adaptive strategies changing dynamically according to the evolution of the epizootic appeared feasible.

## Introduction

Foot and mouth disease (FMD), often considered a prototype of epizootic disease, is an infectious disease affecting cloven-hoofed animals, in particular cattle, sheep, pigs and goats. FMD represents a major concern for developed countries, because of its impact on trade and the economic losses that may result of it. Following the successes obtained for Rinderpest, the Food and Agriculture Organization of the United Nations (FAO) and the World Organisation for Animal Health (OIE) recently launched a global strategy to eradicate FMD worldwide. In many countries of the European Union (EU), annual vaccination of cattle against FMD was performed from the 1960's. This measure, associated with the stamping-out of infected farms and the restriction of animal movements in infected areas, allowed the successful control of all endemic foci at the end of the 1980's. After a risk-benefit analysis, this success led the EU to prohibit all vaccination after 1991. Emergency vaccination remained however possible for epizootic control, but this tool was not used in recent epizootics: member states preferred using preemptive cull of dangerous contacts herds (except for the Dutch specific context, in 2001). However, the development of new vaccines and serological DIVA tests (that can differentiate vaccinal and natural immunity) could allow extending the use of vaccine [1]–[3]. Besides basic mandatory measures, fixed by EU regulations (stamping-out and disinfection in infected farms, restrictions of animal movements…), the panel of available measures for the design of FMD control strategies is relatively rich and may combine or not, according to the susceptible species, preemptive cull, suppressive vaccination (vaccinated animals are culled at the end of the epizootic like in Dutch in 2001) and protective vaccination (vaccinated animals are not culled but are subjected to movement restrictions).

Simulation models have been used to analyze the influence of the environment (e.g. farms density and aggregation) and farming practices (e.g. those that induce movements of animals between farms) on the spread of an FMD epizootic, and the efficacy of control measures. For example, Green *et al.* [4] analyzed the respective impact of animal movements and of local spread on the size and geographic extent of an epizootic; and Orton *et al.* [5] investigated the efficacy of movement control measures. Tildesley *et al.* [6] compared emergency ring vaccination strategies in various epidemiological contexts and under logistical constraints. A similar study was devoted to the comparison of preemptive culling strategies [7]. Comparison of control strategies against simulated FMD epizootics allows determining optimal strategies according to their epidemiological and/or economic impacts [8], [9]. Studied impacts may be first epidemiological: in the study of Martinez *et al.* [10], for example, depopulation and vaccination of farms in contact with outbreaks decreased the number of infected herds, compared to the stamping-out of outbreaks alone. This result was confirmed by Tildesley *et al.* [11], who also showed that preemptive slaughter alone was sometimes unable to control an epizootic. Some studies consider at the same time the economic and epidemiologic consequences FMD epizootics: Schoenbaum *et al.* [12], for example, showed that the early ring vaccination resulted in lower government costs and outbreak duration in fast-spreading scenarios. However, studied impacts are also often only economic [13]: for Garner *et al.* [14], the stamping-out strategy was, on average, the less costly, while Tomassen *et al.* [15] showed that the implementation of alternative strategies (ring-vaccination, ring-culling) could reduce economic impacts. Studied economic impacts may be limited to direct costs (e.g. public costs) or also analyze the impact of an epizootic (and of control measures) on the whole economy (indirect costs, [16], [17]). Indeed, the consequences of FMD epizootics are not limited to the agricultural sector or to agro-food industries; other economic sectors are affected such as tourism [18]. Part of the economic impacts result from the reactions of consumer (or, more generally, of public opinion) against the control measures [19]. More generally, FMD epizootics may have a social impact due to the reactions of public opinion against the control measures, as reported by media. This may lead, for example, to the mobilization against the massive slaughter of animals induced by preemptive slaughter strategies. Even if the problem of the social acceptability of massive slaughter is obvious, the social impact of this type of strategy is not considered in literature, except in some studies on the 2001 epizootic that underlined the importance of public opinion [20], [21].

Decision-making for the control of epizootic diseases does not always obey a pure scientific rationality, based on economic or epidemiological criteria. These decisions remain basically political: in France, in 2007, the government chose to focus the vaccination efforts against bluetongue in the areas that had been the most severely affected the preceding year. This decision was clearly not optimal on an epidemiological or economic point of view; however, it allowed satisfying the breeders who had suffered most the preceding year. Rationalizing the choice of a control strategy against FMD epizootics should take into account several criteria in the decision-making process: the direct economic impact (e.g. public costs), the indirect economic impact (e.g. the export losses for agro-food industries, the shortfall for the tourism sector), and social impacts that may induce a distrust of public control of animal diseases.

The objective of this study was to analyze the choice of a FMD control strategy as a decision-making process in which the decision-maker is influenced by several lobbies, whose interests may be divergent. These lobbies take an active part in the decision-making process and are considered stakeholders in this process. The choice made by the decision-maker depends on the relative weights of these stakeholders (or, alternatively, on the relative influence of the corresponding lobbying groups). Three stakeholders were considered: the government (that represents public finances), the public opinion, and the agro-food industries (that represents the economic sectors directly or indirectly affected by FMD epizootics). We determined how, in France, the optimal control strategy would vary according to the relative weights of stakeholders and to the perception of risk by the decision-maker (either risk-neutral or risk-averse). Two geographic levels were compared for the decision-making process: a single decision taken at the national level, or independent decisions taken in each region. We finally analyzed the feasibility of adaptive decision-making processes that could allow adapting the choice of the control strategy depending on the date and place where the epizootic starts, or depending on the evolution of the epizootic over time.

## Materials and Methods

### Disease transmission, detection and control models

We used the simulation model of FMD epizootics developed by Rautureau *et al.* [22]. This tool allows simulating realistic FMD epizootic dynamics in France, based upon actual data on French holdings (N = 390,565 including farms, markets, dealers and slaughterhouses) and on animal movements of cattle [23] and swine [24]. The basic unit was the holding, consisting of at most three mono-specific animal batches: cattle, small ruminants and swine. Five individual exclusive health states were considered: exposed and susceptible (denoted S), infected but not infectious (denoted L), subclinically infectious (denoted I), clinically infectious (denoted J) and immune (denoted R). Inside a holding, we assumed that animals of a given species batch were exposed to three forces of infection: the within-batch force of infection, the between-batch force of infection and the environmental force of infection. The latter two forces were based upon the within-batch force of infection, decreased according to species-specific biosecurity parameters, which represented the levels of separation between species in usual farm organization and management. Three modes of transmission between holdings were considered: live animal movements (inducing potentially nationwide epizootics), direct transmission by contacts between herds on pastures; and indirect transmission by contaminated vehicles, materials or fomites. Animal movements were simulated according to records stored in a national database. The health state of animals moved from one holding to another was assumed to be random. The direct between-herd force of infection was modelled as a fraction of the within-herd force of infection (decreased by a biosecurity parameter), whereas the indirect between-herd force of infection was modelled as a fraction of the within-herd environmental force of infection (also decreased by a biosecurity parameter). The disease detection model was based upon an explicit representation of the awareness of farmers and veterinarians (i.e. their ability to recognize and report FMD cases) that evolved dynamically when these actors observe diseased animals [22]. Two categories of actors were assumed to be involved in disease surveillance: the farmer who manages the herds within a holding, and the veterinarian responsible for several holdings in a given area. In the model, two events contributed to a disease report: (i) the farmer observes clinical signs and decides whether it is necessary to call the veterinarian, (ii) the veterinarian recognizes the disease and reports a suspected FMD case to the authorities. The probabilities of these events occurring were modeled according to an actor-specific awareness level. Actor awareness was represented by a state variable that increased daily, according to the observations of the actor during the day. Therefore, the time to detection of the first case was not a parameter of the model but an output variable, the value of which resulted from the dynamics of the disease-reporting process. Parameterization of the disease transmission and detection models varied according to the species of the affected animals: infected cattle and swine are known to be highly contagious and to exhibit clear clinical signs, whereas infected small ruminants are known to be less contagious, clinical signs being also lighter. Parameter values were fixed based on literature values or on expert opinions (transmission and detection parameters were fixed using the Delphi method) [22].

The disease control model was based on the stamping-out (SO) strategy: slaughter and disinfection of reported outbreaks, epidemiological inquiry (including contact tracing), and movement restriction in contact herds and in a 10 km radius area around reported outbreaks. Six supplementary control strategies were defined (Table 1) that combined preemptive slaughter and emergency vaccination. Preemptive slaughter corresponded to the slaughter of all herds in a radius of 1 km around reported outbreaks, and of herds identified “at risk” by epidemiological enquiries conducted in outbreaks. Vaccination was either suppressive (the vaccinated animals were culled at the end of the epizootic and their carcasses were destroyed) or protective (the vaccinated animals were not culled at the end of the epizootic but were subjected to movement restrictions). In three of these control strategies, measures were species-specific and varied according to the economic value of the animals (high for cattle and breeding pigs *vs* low for small ruminants and non-breeding pigs, Table 1). To ensure the completion of simulations, it was assumed that, whatever the control strategy, an emergency protective vaccination campaign (PV control strategy in Table 1) was undertaken if the number of reported outbreaks exceeded 500. Such epizootics are termed below “uncontrolled” epizootics, as opposed to “controlled” epizootics, for which no systematic vaccination was necessary. The post-epizootic serological surveys (necessary to FMD-free status recovery) were simulated according to EU regulations (Council directive 2003/85/EC). Human and material resources dedicated to control measures were considered limited, with a maximum of 3 farm depopulations (reported outbreaks having priority over preemptive culls), 5 disinfections and 5 epidemiological inquiries (in affected farms) per day and department; 1000 vaccinated animals per day and veterinarian; and 8000 serological analyses per day. If several departments were affected, their resources were pooled. Resources dedicated to each control task (depopulation, disinfection, epidemiological inquiries, vaccination) were considered independent (because of the different qualifications required for each task), and were thus not prioritized.

**Table 1:** Simulated FMD control strategies.

Code	Name	Preemptive slaughter		Emergency vaccination		
		Species	Radius	Species	Radius	Cull
SO	Stamping-out	-	-	-	-	-
PV	Protective vaccination	-	-	All	10 km	No
PS	Preemptive slaughter	All	1 km	-	-	-
SV	Suppressive vaccination	-	-	All	1 km	Yes
SPV	Selective protective vaccination	-	-	Cattle, breeding pigs	10 km	No
SPS	Selective preemptive slaughter	Small ruminants, non-breeding pigs	10 km	-	-	-
SPSV	Selective preemptive slaughter and protective vaccination	Small ruminants, non-breeding pigs	1 km	Cattle, breeding pigs	10 km	No

Note: Whatever the strategy, basic control measures are applied: slaughter and disinfection of reported outbreaks, contact tracing, movements restriction in contact herds and in a 10 km radius area around reported outbreaks.

### Quantification of epizootic impact for stakeholders

The indicator of epizootic impact for government was the total public expense, based on the following unitary costs [22]:

- depopulation and disinfection operations, compensations paid to farmers: 2,500€ per animal for cattle, 900€ for breeding pigs, 500€ for non-breeding pigs and for small ruminants;
- vaccine and vaccination operations: 5.8€ per vaccinated animal;
- laboratory analyses: 15.4€ per test.

The indicator chosen to quantify the impact of an epizootic on public opinion was the total number of slaughtered herds (either in reported outbreaks, or in herds submitted to preemptive slaughter). Finally, the impact of an epizootic on agro-food industries was quantified by the total export losses caused by bans. Assuming that the OIE zoning principle would be applied using the NUTS (Nomenclature of Territorial Units for Statistics) level 2 (i.e. the 21 French administrative “regions”), and based on OIE and EU regulations, the duration and extent of export bans were fixed as follows:

- exports to EU countries: ban on the exports of meat and dairy products originating from each of the affected regions, ending one week after the completion of the post-epizootic serological surveys,
- exports to non-EU countries: ban on the exports of meat and dairy products originating from each of the affected regions, ending 3 months after the last depopulation if no emergency vaccination is performed, and 6 months after the last depopulation or vaccination otherwise.

For each region, daily export losses that would be caused by an export ban were estimated using the 2010 annual statistics. Meat and dairy products export statistics were obtained from the national institute of statistics and economic studies (INSEE) for 2010 and for each French region, according to the destination of exports: EU *vs* non-EU countries. For a given epizootic, the total export losses were the product of the total daily exports from the affected regions to EU and non-EU countries, by the corresponding ban durations.

### Decision making

For each stakeholder, the choice of a control strategy was modelled according to the perception of risk by the decision-maker. Risk-neutral decision-makers were defined as decision-makers who base their decision on the average of the predicted impact: they would thus choose the control strategy associated with the lowest mean impact. Conversely, risk-averse decision makers rather want to avoid severe epizootic impacts, even if the mean impact is not the lowest. They were defined as decision-makers who base their decision on the variance of the predicted impact: they would thus choose the control strategy associated with the lowest standard deviation of the epizootic impact.

Epizootic impacts (public costs, slaughtered herds and export losses) were first separately computed for each of the three stakeholders (respectively: government, public opinion and agro-food industries) and the optimal control strategy was determined for risk-neutral and risk-averse decision-makers. However, in a real situation, the decision would probably not favour a single stakeholder, but rather result from a compromise between them. Such a collective decision was modelled as a multi-criteria decision, using a simple additive method. Each stakeholder-specific impact was first recalibrated as follows: *X_i,s_* = (*x_i,s_*−*x_SO,s_*)/*x_SO,s_*, where *X_i,s_* is the recalibrated impact of the *i*th simulated epizootic for the stakeholder *s*; *x_i,s_* is the simulated impact of the epizootic *i* for the stakeholder *s*; and *x_SO,s_* is the mean impact of a simulated epizootic for stakeholder *s*, when the basic and mandatory stamping-out (SO) strategy is applied. The recalibrated impact of the epizootic *i* (*X_i,s_*) thus represented the deviation from the mean impact when the stamping-out strategy was applied, the unit being also the average impact when the SO strategy was used.

The “collective impact” *Y_i_* of a the *i*th simulated epizootic was then defined as a linear function of the three recalibrated impacts: *Y_i_* = *w*_g_ *X_i,g_*+*w*_p_ *X_i,p_*+*w*_a_ *X_i,a_*, where the subscripts *g*, *p* and *a* correspond to the government, the public opinion and the agro-food industry, respectively; and *w*_g_, *w*_p_ and *w*_a_ are the weights of the three stakeholders in the collective decision; or, alternatively, those of the corresponding lobbies in the decision-making process. Finally, for a given triple (*w*_g_, *w*_p_, *w*_a_), the optimal strategy was the one that minimized the mean (for a risk-neutral decision-maker) or the variance (for a risk-averse decision-maker) of the collective impact.

### Optimal decision-making for fixed control strategies

**Figure 1:**
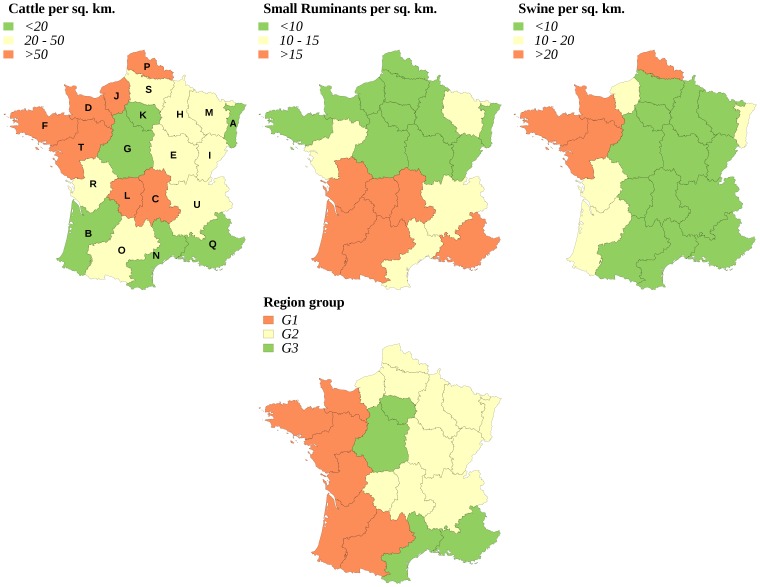
Typology of French regions according to the animal densities of cattle, swine and small ruminants. Group G1: high cattle and pig density (upper class), high density of small ruminants (upper class) associated with a low or medium cattle and swine density. Group G2: high or medium density of cattle and/or swine associated with a similar or lower density of small ruminants (same class or lower class). Group G3: low densities of cattle and swine. Capital letters on the first map are used to identify the French regions: A: Alsace, B: Aquitaine, C: Auvergne, D: Basse-Normandie, E: Bourgogne, F: Bretagne, G: Centre, H: Champagne-Ardenne, I: Franche-Comté, J: Haute-Normandie, K: Ile-de-France, L: Limousin, M: Lorraine, N: Languedoc-Roussillon, O: Midi-Pyrénées, P: Nord-Pas-de-Calais, Q: Provence-Alpes-Côte d'Azur, R: Poitou-Charentes, S: Picardie, T: Pays-de-Loire, U: Rhône-Alpes.

The breeding structures are known to show strong regional variations in France (Fig. 1), and large variations of epizootic impacts were expected according to the region in which the virus was introduced. Simulations were thus stratified by region. Fifty herds were randomly selected among those housing at least one susceptible animal (cattle, small ruminant or pig) in each of the 21 French regions, as well as a random calendar date for virus introduction (a single animal being initially infected, the species of which was randomly chosen). In the following, we define a scenario as a unique situation corresponding to a herd in which the virus is initially introduced, a date for this introduction, and a control strategy. A total number of 7,350 scenarios were thus defined (50 herds and dates of virus introduction, 21 regions, and 7 control strategies). In this part of the study, the aim was to quantify the average impact of FMD epizootics (according to the control strategy and to the region where the virus is initially introduced) rather than the variability of the impacts for a given scenario (as defined above). Therefore, a single simulation was run for each scenario (*i.e.* 50 per region). In these simulations, once started, FMD epizootics could either remain confined to the region where the virus had been introduced, or spread to the neighbouring areas, or further afield. For a given simulation and a given impact, impact value was the total of the impact in the region where the virus had been introduced, plus the impacts in the other affected regions. For each region and each control strategy, we computed the average impact of simulated epizootics starting in that region (and possibly affecting other regions). In order to compute the country-level mean impact of an FMD epizootic, an assumption had to be made about the risk of virus introduction in each region. We assumed that region-specific risks of introduction were proportional to the number of herds in each region. These numbers were thus used as weights to calculate the average impact of an FMD epizootic in France, as well as the corresponding variance. Optimal strategies were identified for each stakeholder and for the two types of risk perception by decision-makers. Collective impacts were computed for varying combinations of stakeholder weights: the weight of the government (*w_g_*) was fixed to a constant value of 1 whereas the weights of agro-food industry (*w_a_*) and of public opinion (*w_p_*) independently varied between 10^−3^ and 10^3^. The corresponding optimal strategies were separately determined for risk-neutral and for risk-averse decision-makers, and the variations of the optimal strategy according to stakeholder weights were plotted.

### Feasibility of decision-making for adaptive control strategies

Two types of adaptations were considered: the choice of a fixed strategy according to the place where the epizootic starts (the “geographic” adaptation), and the dynamical adaptation of the strategy according to how the epizootic evolves (the “time” adaptation). In both cases, we decomposed the variability of impacts into an explained part (by geography and/or by time), and an unexplained part that results from the intrinsic variability of epizootics. The feasibility of adaptive control strategies was considered proportional to the explained part of the impact variability. This part of the study was restricted to the three regions where livestock density was the highest (regions D, F and T, Fig. 1).

To study the feasibility of a geographic adaptation of the control strategy, 350 scenarios were defined: 50 herds were randomly chosen in regions D, F ant T, as well as random dates for virus introduction, and the 7 control strategies were associated to each of these 50 herds. Because the aim of this part of the study was to analyze the variability of epizootic impacts, 30 simulations were run for each scenario (changing the seed of the random number generator). A total of 10,500 simulations were thus executed and the impacts were computed for each of the three stakeholders. The variability of the log-transformed impacts was analyzed separately for each control strategy, using linear mixed models in which the scenario (coded as a single categorical variable) was treated as a random effect, and without fixed effects. The part of variance explained by the scenario was computed, the intrinsic variability of epizootics was the residual variance.

To study the feasibility of a time adaptation of the control strategy, 70 scenarios were defined, that combined the 7 control strategies and 10 herds. These were randomly chosen in regions D, F and T, and a randomly chosen virus introduction date was associated with each of them. A total of 5^5^ simulations were run for each scenario as follows. Starting from the date of virus introduction, 5 simulations were first run (changing the seed of the random number generator) and stopped when the first infected premise was detected, at day D0. Each of these 5 situations at D0 was successively used as the starting situation for 5 new simulations, which were started at D0 and stopped at D7. A total of 25 distinct situations were thus obtained at D7. The same operation was recursively performed at D14, D28 and D56. Globally, a total number of 218,750 (70 scenarios×5^5^ simulations) were thus executed and the impacts were computed for each of the three stakeholders. The log-transformed impacts were separately analyzed for each control strategy, using linear mixed models. The scenario (place and date where the epizootic starts) was treated as a random effect. The D0–D7, D7–D14, D14–D28, and D28–D56 evolutions were treated as recursively nested random effects, the D0–D7 evolution being also nested in the initial conditions. There was no fixed effect. The part of variance explained by the D0–D7, D7–D14, D14–D28 and D28–D56 evolutions were computed; the intrinsic variability of epizootics was the residual variance.

## Results

### General results

**Figure 2:**
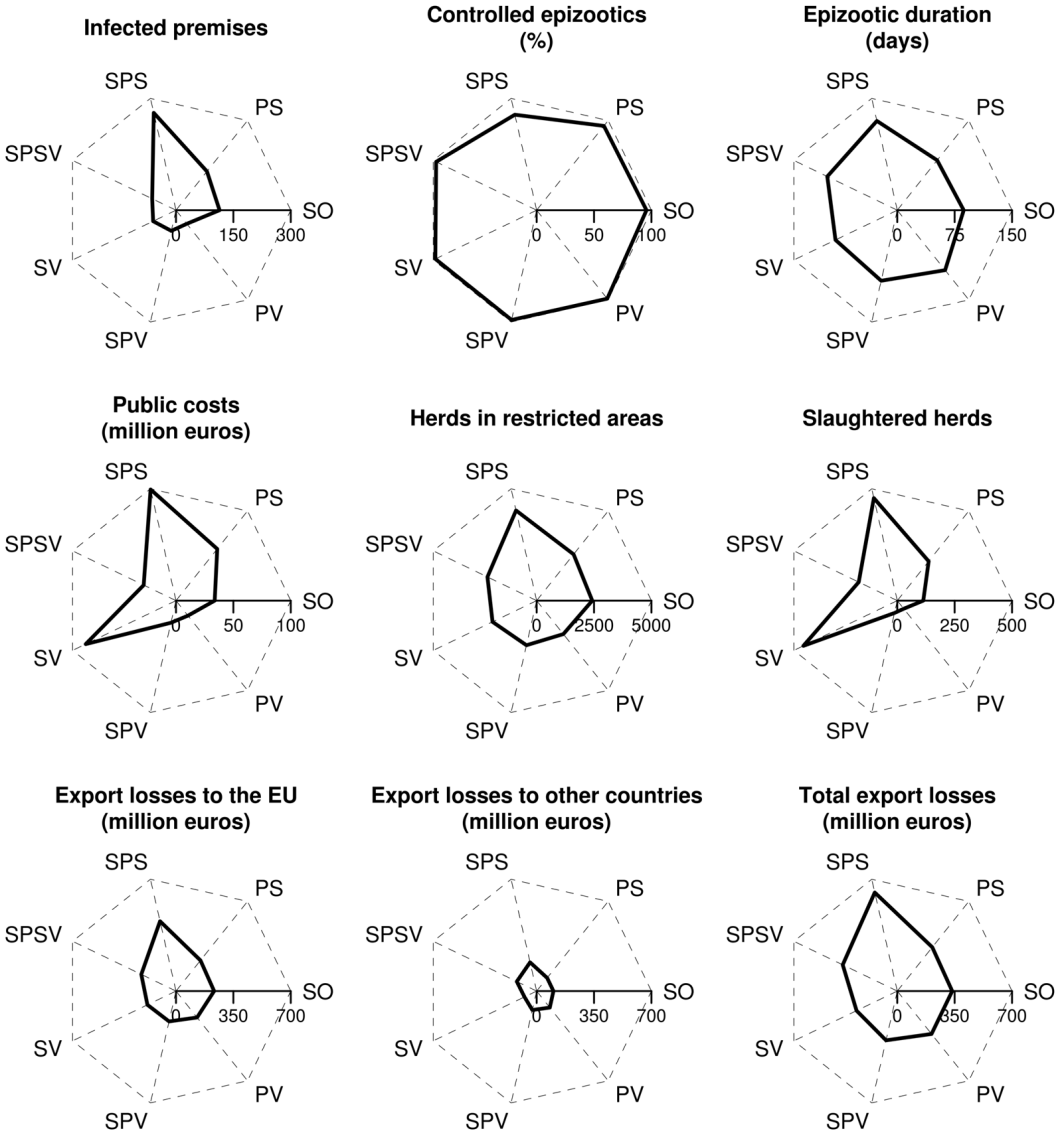
Average impact of control strategies on simulated FMD epizootics in France. SO: basic stamping-out strategy, PV: protective vaccination, PS: preemptive slaughter, SPV: selective protective vaccination, SPS: selective preemptive slaughter, SPSV: selective preemptive slaughter and protective vaccination, SV: suppressive vaccination.

The vast majority of simulated epizootics could be controlled without the help of an emergency protective vaccination campaign. At the national level, the proportion of controlled epizootics was high whatever the control strategy (Fig. 2): 96% for the basic stamping-out strategy and higher for the strategies with vaccination, reaching 99% for the protective vaccination (PV) strategy. The worst strategy was the selective preemptive slaughter (SPS) strategy: an emergency protective vaccination campaign was necessary to epizootic control in 15% of simulations. It was also the strategy for which the average number of infected premises was the highest, whereas this average number was the lowest for the strategies with vaccination. The national-level average duration of epizootics (number of days between the first outbreak report and the end of the post-epizootic surveys) and their average geographic extent (represented by the number of herds in the restricted areas) were similar for most of the strategies, and a little higher for the SPS strategy (Fig. 2.). The average number of slaughtered herds was high for the SPS and for the suppressive vaccination (SV) strategies, but for very different reasons: because of a greater mean number of infected premises for the SPS strategy, and because of the slaughter of vaccinated animals for the SV strategy. The corresponding compensations, paid to farmers, represented the main public costs, which were thus on average higher for the SPS and SV strategies (Fig. 2). Average total export losses were higher for the SPS strategy, and lower for the strategies with vaccination, despite the longer ban durations when protective vaccination is used. This was due to a shorter average duration of epizootics when vaccination was used, associated to a lower geographic extent of epizootics (the number of regions submitted to export bans being then lower). Furthermore, the long duration of bans mainly affected exports to non-EU countries, which represented only 22% of exports for meat products, and 23% for dairy products. Most of the export losses were thus due to EU bans (Fig. 2).

### Decisions of national-scope

When the decision was taken at the national-level, whatever their perception of risk, decision-makers wishing to minimize the public costs or the number of slaughtered herds chose the protective vaccination strategy (Table 2). Decision-makers wishing to minimize export losses also chose a strategy based upon vaccination: the suppressive vaccination strategy for risk-neutral decision-makers and the selective protective vaccination strategy for risk-averse decision-makers (Table 2). The corresponding export losses were, on average, €33 million higher for a risk-averse than for a risk-neutral decision-maker. However, in 21% of epizootics (on average, at the national level), the export losses were higher for the risk-neutral than for the risk-averse decision-maker.

**Table 2:** Average impact of simulated FMD epizootics according to the risk perception of decision makers and to the geographic level at which the control strategy is chosen and applied.

Impact	Decision-makers risk perception	Geographic decision level		National/Regional
		National mean (sd^1^)	Regional mean (sd)	Ratio for mean (sd)
Public costs^5^	Risk neutral	16.7 (33.7)^2^	16.0 (33.5)	0.95 (1.00)
	Risk averse	16.7 (33.7)^2^	16.3 (33.4)	0.97 (0.99)
Export losses^5^	Risk neutral	275.7 (502.9)^3^	250.0 (457.2)	0.91 (0.91)
	Risk averse	308.3 (456.2)^4^	277.4 (430.8)	0.90 (0.94)
Slaughtered herds	Risk neutral	45.2 (107.0)^2^	45.0 (106.8)	1.00 (1.00)
	Risk averse	45.2 (107.0)^2^	45.3 (106.6)	1.00 (1.00)

1 Standard deviation.

2 Optimal strategy: protective vaccination.

3 Optimal strategy: suppressive vaccination.

4 Optimal strategy: selective protective vaccination.

5 Million €.

Note: National level: a single decision is taken and applied to the whole country. Regional level: specific decisions are taken and applied in each region.

**Figure 3:**
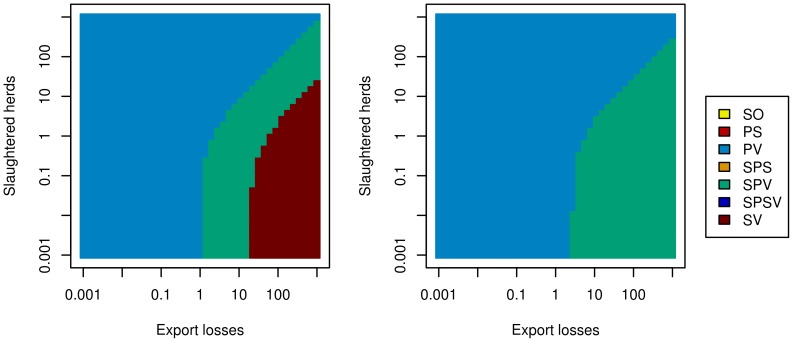
Optimal national control strategies according to the risk-perception and to the respective weights of stakeholders. Optimal control strategy decided at the national level by a risk-neutral decision-maker (left) or by a risk-averse decision-maker (right), according to the respective weights of stakeholders in decision-making: government (indicator: public costs, constant weight of 1), agro-food industry (indicator: export losses, x-axis), public opinion (indicator: number of slaughtered herds, y-axis). SO: basic stamping-out strategy, PV: protective vaccination, PS: preemptive slaughter, SPV: selective protective vaccination, SPS: selective preemptive slaughter, SPSV: selective preemptive slaughter and protective vaccination, SV: suppressive vaccination.

After a collective decision-making process, the chosen strategy remained the protective vaccination strategy if the weight of the government was the strongest, whatever the risk perception of the decision-maker (Fig. 3, lower-left quadrant in both diagrams). It was also the case if the weight of public opinion was the strongest (Fig. 3, upper-left quadrant in both diagrams). Conversely, if the weight of the agro-food industries in the decision process was the strongest (Fig. 3, lower-right quadrant of both diagrams), the optimal strategy became the selective protective vaccination for risk-averse decision-makers, as well as for risk-neutral decision-makers when the relative weight of agro-food industries was moderate. When the relative weight of agro-food industries was high and the decision-maker was risk-neutral, the optimal collective decision became the suppressive vaccination strategy. Finally, when the weight of the government was weaker than that of public opinion and of agro-food industries (Fig. 3, upper right quadrant), the chosen strategy was the protective vaccination when the public opinion had a stronger weight than agro-food industries, and selective protective vaccination or suppressive vaccination otherwise.

### Decisions of regional-scope: decisions taken by each stakeholder

The independent choice of optimal strategies in each region induced a reduction of average national-level public costs of approximately 5%, and a very low reduction of the standard deviation of public costs (Table 2). No significant reduction of the mean and standard deviation was observed for the average number of slaughtered herds. Conversely, a 10% reduction was observed for the export losses, both for the mean value and for the standard deviation.

**Figure 4:**
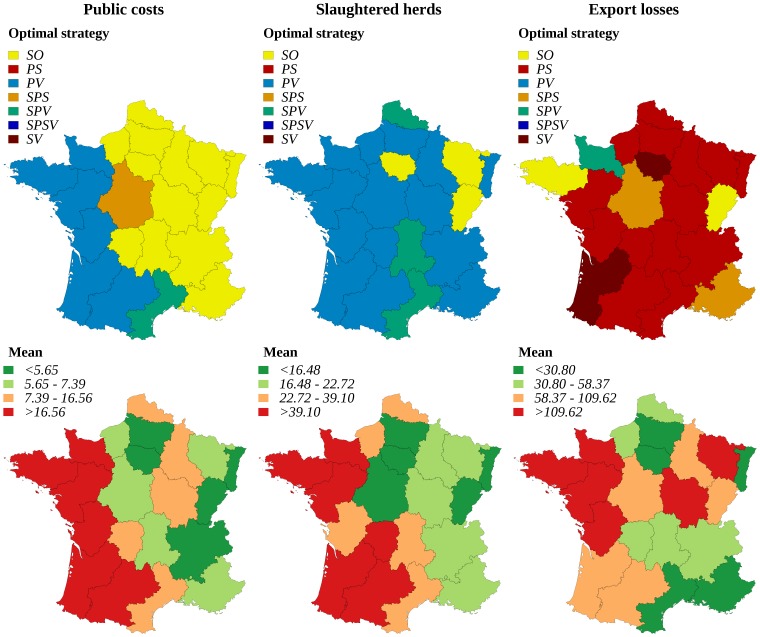
Optimal regional control strategies decided by risk-neutral decision-makers, and corresponding mean impact for each stakeholder. Optimal control strategies decided at the regional level by risk-neutral decision-makers, and corresponding mean impact of FMD epizootics for each stakeholder: government (indicator: public costs), agro-food industry (indicator: export losses), and public opinion (indicator: number of slaughtered herds). SO: basic stamping-out strategy, PV: protective vaccination, PS: preemptive slaughter, SPV: selective protective vaccination, SPS: selective preemptive slaughter, SPSV: selective preemptive slaughter and protective vaccination, SV: suppressive vaccination. Public costs and export losses are given in million €. Class bounds were fixed using the quartiles of the distributions.

**Figure 5:**
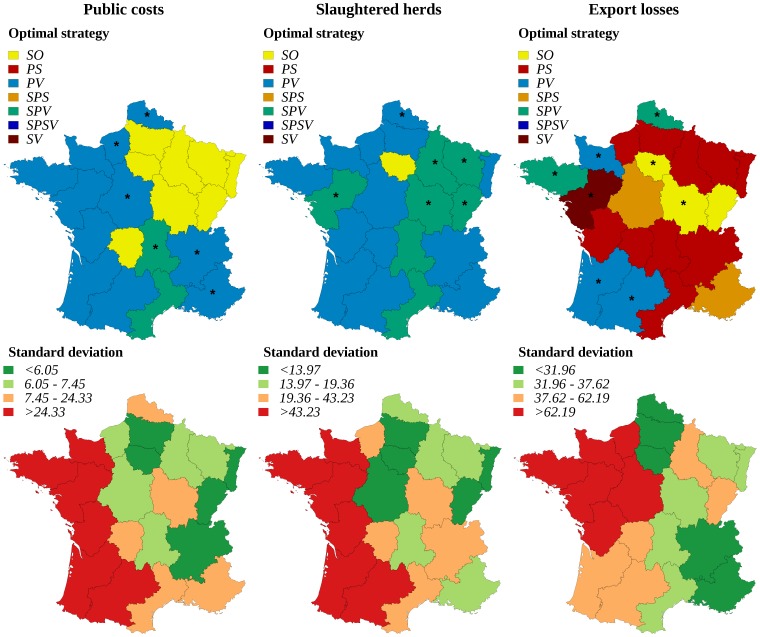
Optimal regional control strategies decided by risk-averse decision-makers, and corresponding standard deviation of the impact. Optimal control strategies decided at the regional level by risk-averse decision-makers, and corresponding standard deviation of the impact of FMD epizootics for each stakeholder: government (indicator: public costs), agro-food industry (indicator: export losses), and public opinion (indicator: number of slaughtered herds). SO: basic stamping-out strategy, PV: protective vaccination, PS: preemptive slaughter, SPV: selective protective vaccination, SPS: selective preemptive slaughter, SPSV: selective preemptive slaughter and protective vaccination, SV: suppressive vaccination. Public costs and export losses are given in million €. Class bounds were fixed using the quartiles of the distributions. Regions where risk-neutral and risk-averse decision-makers disagree are indicated by stars.

For risk-neutral decision-makers (Fig. 4), the strategy allowing minimizing public costs was always based on protective vaccination in western regions where herd density is the highest (e.g. regions D, F and T). It was the basic stamping-out strategy in the other regions (except region G where the selective preemptive slaughter was optimal). To minimize the number of slaughtered herds, the optimal strategy was always based on protective vaccination (PV or SPV), except in three regions for which the stamping-out strategy was preferred. To minimize export losses, the optimal strategy was based on preemptive slaughter (PS or SPS) in 16 of 21 regions, selective protective vaccination (SPV) being optimal in a single region and suppressive vaccination (SV) in two regions. To minimize public costs and slaughtered herds, the optimal strategy was different for risk-averse and risk-neutral decision makers in six regions (indicated by stars in Fig. 5), where the protective vaccination was preferred (PV or SPV). Finally, to minimize export losses, strategies based on protective vaccination (PV or SPV) were more often optimal (5 regions) for risk-averse decision makers, and strategies based on preemptive slaughter (PS or SPS) were less often optimal (12 of 21 regions) (Fig. 5).

### Decisions of regional-scope: collective decision-making

**Figure 6:**
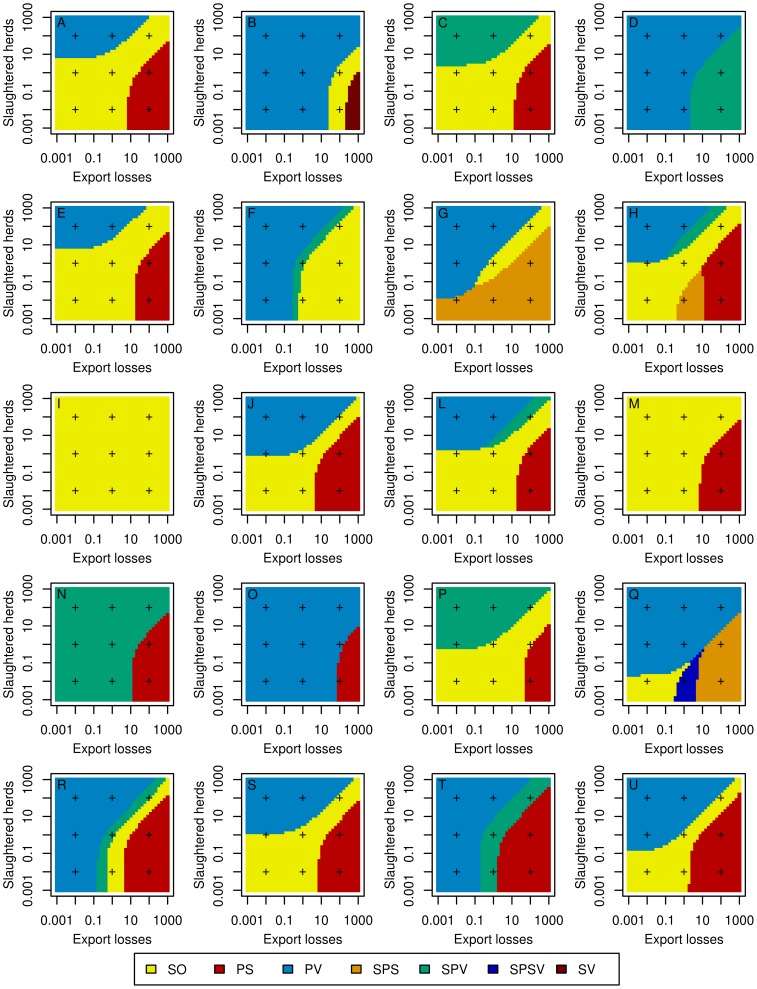
Optimal regional control strategies decided by risk-neutral decision-makers, according to the respective weights of stakeholders. Optimal control strategies decided at the regional level by risk-neutral decision-makers, according to the respective weights of stakeholders in decision-making: government (indicator: public costs, constant weight of 1), agro-food industry (indicator: export losses, x-axis), and public opinion (indicator: number of slaughtered herds, y-axis). SO: basic stamping-out strategy, PV: protective vaccination, PS: preemptive slaughter, SPV: selective protective vaccination, SPS: selective preemptive slaughter, SPSV: selective preemptive slaughter and protective vaccination, SV: suppressive vaccination. Plus signs indicate weights combinations for which maps are drawn in figs. 5a and 5b. Capital letters indicate the region as in fig. 3a.

**Figure 7:**
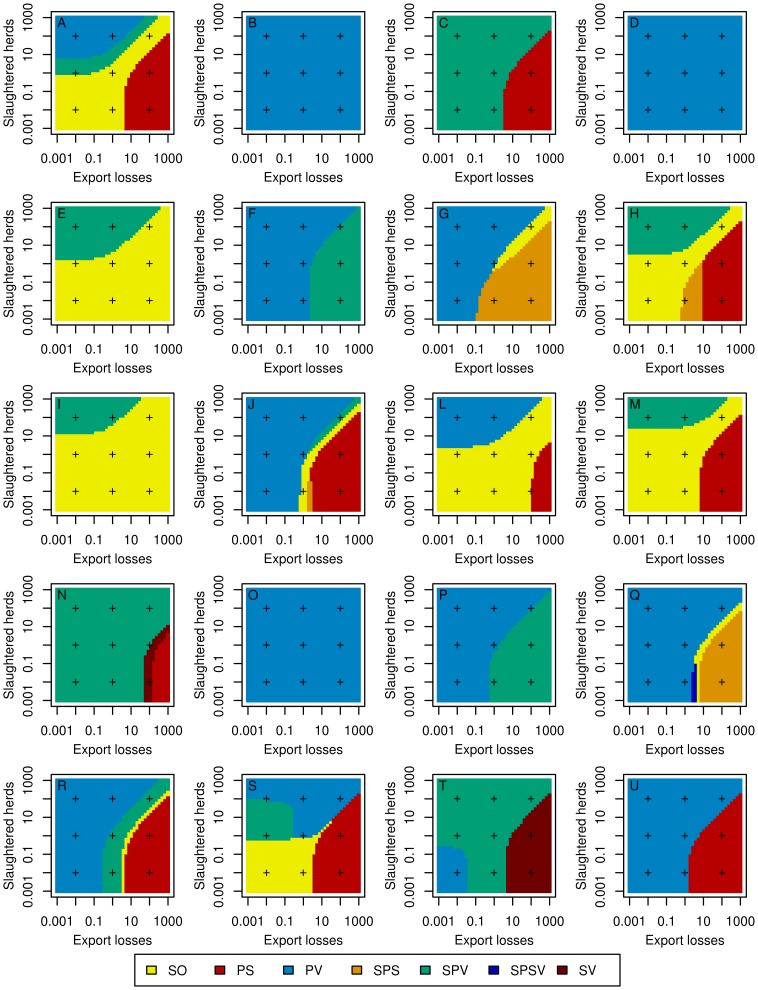
Optimal regional control strategies decided by risk-averse decision-makers, according to the respective weights of stakeholders. Optimal control strategies decided at the regional level by a risk-averse decision-makers, according to the respective weights of stakeholders in decision-making: government (indicator: public costs, constant weight of 1), agro-food industry (indicator: export losses, x-axis), and public opinion (indicator: number of slaughtered herds, y-axis). SO: basic stamping-out strategy, PV: protective vaccination, PS: preemptive slaughter, SPV: selective protective vaccination, SPS: selective preemptive slaughter, SPSV: selective preemptive slaughter and protective vaccination, SV: suppressive vaccination. Plus signs indicate weights combinations for which maps are drawn in figs. 5a and 5b. Capital letters indicate the region as in fig. 3a.

**Figure 8:**
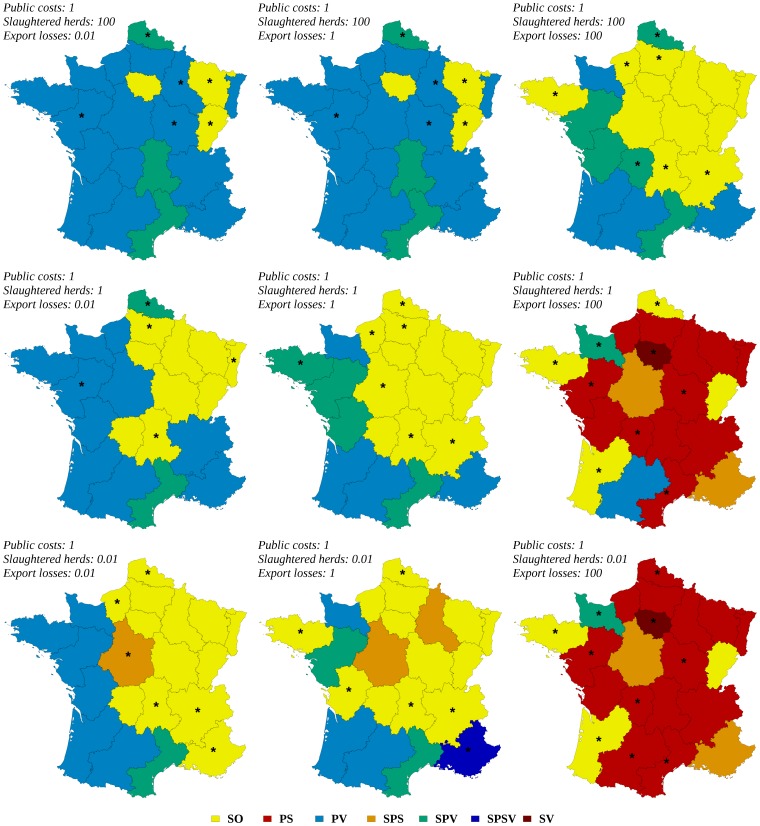
Optimal regional control strategies decided by risk-neutral decision-makers, for specific combinations of stakeholders weights. Optimal control strategies decided at the regional level by risk-neutral decision-makers, for specific combinations of stakeholders weights in decision-making: government (indicator: public costs), agro-food industry (indicator: export losses), and public opinion (indicator: number of slaughtered herds). SO: basic stamping-out strategy, PV: protective vaccination, PS: preemptive slaughter, SPV: selective protective vaccination, SPS: selective preemptive slaughter, SPSV: selective preemptive slaughter and protective vaccination, SV: suppressive vaccination. Regions where risk-neutral and risk-averse decision-makers disagree are indicated by stars.

**Figure 9:**
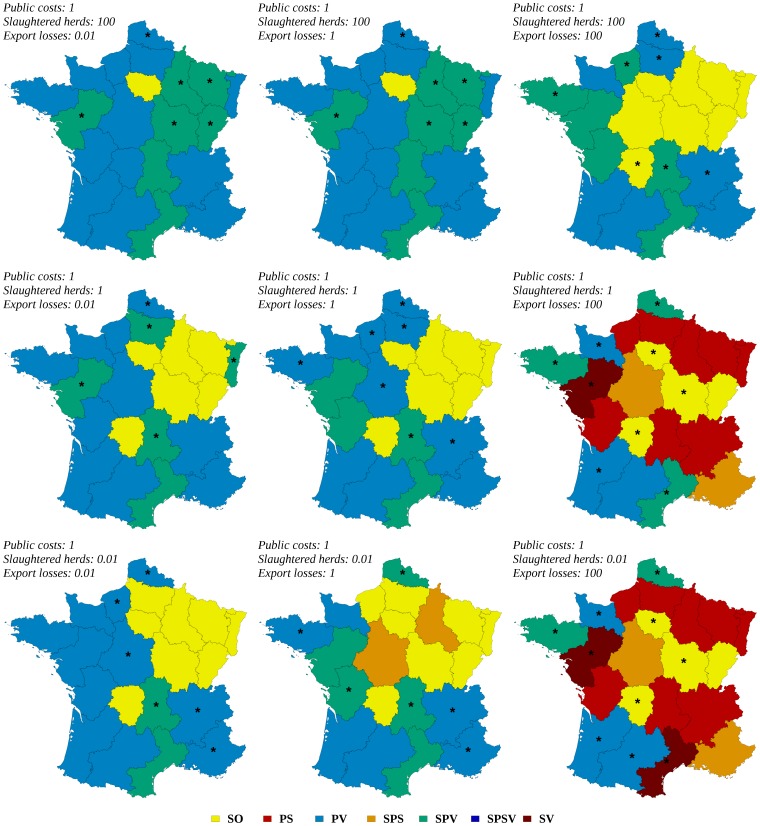
Optimal regional control strategies decided by risk-averse decision-makers, for specific combinations of stakeholders weights. Optimal control strategies decided at the regional level by risk-averse decision-makers, for specific combinations of stakeholders weights in decision-making: government (indicator: public costs), agro-food industry (indicator: export losses), and public opinion (indicator: number of slaughtered herds). SO: basic stamping-out strategy, PV: protective vaccination, PS: preemptive slaughter, SPV: selective protective vaccination, SPS: selective preemptive slaughter, SPSV: selective preemptive slaughter and protective vaccination, SV: suppressive vaccination. Regions where risk-neutral and risk-averse decision-makers disagree are indicated by stars.

The result of a collective decision-making process showed various and sometimes complex patterns according to the region and to the relative weights of the three stakeholders (Fig. 6 and 7). In region I (see Fig. 1 for the location of regions), for a risk-neutral decision-maker, the optimal strategy was always the stamping-out strategy, whatever the weights of stakeholders (Fig. 6). In region D, the optimal strategy was always the protective vaccination strategy (whatever the relative weights if stakeholders) for a risk-averse decision-maker (Fig. 7). More complex patterns were obtained in other regions such as in region H: for a risk neutral decision-maker, five of the seven studied strategies could become optimal according to the relative weights of stakeholders (Fig. 6). Figures 8 and 9 map the optimal strategy for a risk-neutral and a risk-averse decision-maker respectively and for specific combinations of stakeholder weights (indicated by crosses in figures 6 and 7). The region-specific decision patterns of risk-neutral decisions-makers shown in Fig. 6 could be classified in 3 groups:

- Group G1 (regions B, D, F, O, R and T): the decision pattern was divided in two areas. The protective vaccination (PV) strategy was optimal when the weight of the government or of the public opinion exceeded that of the agro-food industries. It was replaced by another strategy when the agro-food industries dominated: selective protective vaccination (region D), stamping-out (region F), suppressive vaccination (region B) and/or preemptive slaughter (regions O, R, T). The regions that compose this group were characterized by either a high density of cattle and swine (regions D, F and T), or a high density of small ruminants associated with a low or medium density of cattle (regions B, O, R, Fig. 1).
- Group G2 (regions A, C, E, H, J, L, P, S and U): the decision pattern was divided in three areas. The stamping-out strategy was optimal when the government's weight was the strongest, or when the agro-food industries and the public opinion had similar weights. When the public opinion dominated the decision, a vaccination-based strategy was optimal, whereas if the weight of agro-food industries was the strongest, the preemptive slaughter strategy was preferred. The regions that compose this group were characterized by high or medium cattle and/or swine densities, the density of small ruminants being always lower than the density of cattle (Fig. 1). Regions M and I also shared these characteristics.
- Group G3: regions G, N and Q showed various decision patterns according to stakeholders' weights. They were characterized by low cattle and swine densities, possibly associated with high (region Q) or medium (region N) densities of small ruminants.

This classification of decision patterns remained globally valid for a risk-averse decision-maker (Figs. 7 and 9), with a wider use of vaccination-based strategies, and a less frequent use of preemptive slaughter. In the G1 group, the protective vaccination strategy was always optimal for risk-averse decision-makers in regions B, D and O, whereas the decision pattern remained similar to that of risk-neutral decision-makers in the other regions (F, R and T). The decision pattern was also conserved for the G2 group in regions A, H, L, M and S. When agro-food industries dominated the decision, preemptive slaughter was sometimes replaced by the stamping-out strategy or by selective protective vaccination (regions E and P). Similarly, when the government's weight was the strongest, the stamping-out strategy was sometimes replaced by a strategy based on protective vaccination (PV or SPV in regions C, J, P and U). For the G3 group, the decision patterns obtained for risk-averse decision-makers were similar to these of risk-neutral decision-makers.

### Feasibility of decision-making for adaptive control strategies

The initial conditions (time and place where the simulated epizootic begins) always explained at least half of the variance of impacts, whatever the stakeholder and the control strategy. The intrinsic variance of epizootics (the residual variance) remained however significant, the lowest value (*i.e.* the situation with the best predictability) being 25% of the total variance for export losses with the protective vaccination strategy (Table 3). Besides the effect of the initial conditions, when the evolution of the epizootic was taken into account, the proportion of residual variance was the lowest (the predictability being thus better) for the protective vaccination strategy and for the selective protective vaccination strategy (Table 4). After removal of the variance attributable to the initial conditions and with the PV strategy, the proportion of residual variance was 2% for the public costs and for the number of slaughtered herds. It was 9% for the export losses, the proportion of residual variance being globally higher for this type of impact. The highest proportion of residual variance was observed for the selective preemptive slaughter and for the stamping-out strategies: >10% for the public costs and for the number of slaughtered herds, and >30% for the export losses (Table 4). For the public costs and for the number of slaughtered herds, the proportion of variance attributable to the evolution of the epizootics during successive periods was often higher for the first two periods: D0–D7 and D7–D14. The opposite situation was observed for the export losses, the last two periods having a stronger impact on the overall variance: D14–D28 and D28–D56 (Table 4).

**Table 3:** Feasibility of fixed control strategies adapted to the time and place where a simulated FMD epizootic begins.

	Control strategy						
	SO	PS	PV	SPS	SPV	SPSV	SV
Direct costs							
Initial conditions	1.4 (52%)	1.7 (59%)	0.8 (55%)	3.3 (58%)	0.8 (53%)	1.3 (58%)	1.2 (53%)
Residual variance	1.3 (42%)	1.2 (41%)	0.6 (45%)	2.4 (42%)	0.7 (47%)	0.9 (42%)	1.1 (47%)
Export losses							
Initial conditions	0.4 (60%)	0.6 (64%)	0.3 (75%)	0.8 (60%)	0.3 (68%)	0.3 (68%)	0.3 (58%)
Residual variance	0.3 (40%)	0.3 (36%)	0.1 (25%)	0.5 (40%)	0.1 (32%)	0.2 (32%)	0.2 (42%)
Slaughtered herds							
Initial conditions	1.1 (42%)	1.3 (57%)	0.6 (51%)	1.9 (55%)	0.6 (50%)	0.9 (55%)	1.0 (52%)
Residual variance	1.0 (48%)	1.0 (43%)	0.6 (49%)	1.6 (45%)	0.6 (50%)	0.8 (45%)	0.9 (48%)

Note: The table gives, for a given impact and for a specific control strategy, the parts of variance explained by the initial conditions (place and date where the epizootic begins) and by the intrinsic variability of epizootics (residual variance). Impacts correspond to three stakeholders: government (indicator: public costs), agro-food industry (indicator: export losses), and public opinion (indicator: number of slaughtered herds).SO: basic stamping-out strategy, PV: protective vaccination, PS: preemptive slaughter, SPV: selective protective vaccination, SPS: selective preemptive slaughter, SPSV: selective preemptive slaughter and preventive vaccination, SV: suppressive vaccination. Impact indicators have been log-transformed.

**Table 4:** Feasibility of adaptive control strategies dynamically updated according to the evolution of simulated FMD epizootics.

	Control strategy						
	SO	PS	PV	SPS	SPV	SPSV	SV
Direct costs							
D0–D7 period	0.134 (28%)	0.180 (34%)	0.067 (30%)	0.329 (26%)	0.114 (39%)	0.153 (43%)	0.114 (29%)
D7–D14 period	0.134 (28%)	0.120 (23%)	0.069 (31%)	0.166 (13%)	0.076 (26%)	0.056 (16%)	0.093 (24%)
D14–D28 period	0.094 (19%)	0.111 (21%)	0.059 (26%)	0.282 (22%)	0.063 (22%)	0.079 (22%)	0.090 (23%)
D28–D56 period	0.074 (15%)	0.075 (14%)	0.024 (11%)	0.258 (21%)	0.030 (10%)	0.055 (15%)	0.068 (17%)
Residual variance	0.049 (10%)	0.040 (8%)	0.004 (2%)	0.219 (17%)	0.009 (3%)	0.016 (5%)	0.026 (7%)
Export losses							
D0–D7 period	0.015 (12%)	0.013 (12%)	0.004 (13%)	0.051 (15%)	0.006 (15%)	0.009 (16%)	0.009 (29%)
D7–D14 period	0.015 (12%)	0.017 (15%)	0.005 (18%)	0.030 (9%)	0.006 (15%)	0.006 (11%)	0.011 (12%)
D14–D28 period	0.021 (17%)	0.025 (23%)	0.009 (34%)	0.073 (21%)	0.012 (29%)	0.013 (25%)	0.026 (29%)
D28–D56 period	0.032 (27%)	0.028 (26%)	0.07 (26%)	0.082 (24%)	0.012 (28%)	0.015 (28%)	0.026 (29%)
Residual variance	0.038 (32%)	0.025 (23%)	0.003 (9%)	0.108 (31%)	0.006 (14%)	0.010 (19%)	0.017 (19%)
Slaughtered herds							
D0–D7 period	0.114 (31%)	0.114 (29%)	0.057 (37%)	0.221 (28%)	0.079 (39%)	0.112 (41%)	0.081 (26%)
D7–D14 period	0.081 (22%)	0.081 (22%)	0.036 (23%)	0.116 (14%)	0.047 (23%)	0.043 (16%)	0.074 (24%)
D14–D28 period	0.072 (19%)	0.088 (24%)	0.039 (26%)	0.181 (23%)	0.046 (23%)	0.061 (22%)	0.077 (25%)
D28–D56 period	0.060 (16%)	0.061 (16%)	0.018 (12%)	0.158 (20%)	0.023 (11%)	0.043 (16%)	0.057 (18%)
Residual variance	0.044 (12%)	0.033 (9%)	0.003 (2%)	0.122 (15%)	0.007 (4%)	0.014 (5%)	0.023 (7%)

Note: The table gives, for a given impact and for a specific control strategy, the parts of variance attributable to the epizootic evolution during specific periods, and to the intrinsic variability of epizootics (residual variance). The part of variance imputable to the initial conditions (place and date where the epizootic starts) is not considered here. Impacts correspond to three stakeholders: government (indicator: public costs), agro-food industry (indicator: export losses), and public opinion (indicator: number of slaughtered herds). SO: basic stamping-out strategy, PV: protective vaccination, PS: preemptive slaughter, SPV: selective protective vaccination, SPS: selective preemptive slaughter, SPSV: selective preemptive slaughter and protective vaccination, SV: suppressive vaccination. Impact indicators have been log-transformed.

## Discussion

The choice of a FMD control strategy was analyzed as a collective decision-making process in which three types of stakeholders are involved: the government, the agro-food industries and the public opinion. Seven simulated control strategies, the most realistic to implement, were compared and two geographic levels were considered for the decision scope: national-scope decisions made once for the whole country, and regional-scope decisions independently made in each region. Two types of decision-makers were distinguished according to their perception of risk: risk-neutral and risk-averse decision-makers (risk-seeker decision makers being considered unrealistic for the control of an epizootic disease). The main factor influencing the optimality of a strategy was the type of stakeholder, with opposite effects according to the decision scope: whereas national-scope optimal strategies were based on vaccination whatever the stakeholder, this consensus concealed a strong heterogeneity when the decision scope was regional. Besides the stakeholder and the geographic level, the risk perception by the decision-maker only induced minor variations on the optimal decision. Vaccine was more often used by risk-averse decision-makers and preemptive slaughter less often. However the type of optimal strategy (vaccination or preemptive slaughter) was often the same for risk-neutral and risk-averse decision-makers.

In this study, we used a realistic model of FMD spread, which was forced by actual data about herd populations and animal movements. Differences between species for clinical signs and infectiousness were taken into account. As in other studies [9], [19], [25], the model accounted for the heterogeneity of the within-herd virus diffusion according to the species housed, and to the efficacy of the biosecurity barriers between species inside a farm [26]–[28]. The disease detection process was explicitly represented in the model. Therefore, the time to first detection was not a parameter of the model, but a stochastic output. No correlation was observed between time to detection and epizootic duration or size (number of reported outbreaks). Despite the costs for government were quantified relatively precisely, a simplified cost model was used. For example, losses borne by farmers (e.g. induced by movement restrictions) were not taken into account. A more comprehensive cost model may be found in Backer *et al.* [29]. Herd culls performed for welfare reasons were not included in the model. This could have induced an under-estimation of the social impact of the control strategies. Indeed, the indicator we chose to quantify the social impact was simple. This impact may also be influenced, for example, by the total duration of the epizootic and the extent of the area submitted to restrictions of animal and human movement. It may also be linked to the impact on agro-food industries, which could induce a slowdown in economic activity. The indicator we chose for the impact on agro-food industries (export losses) was also rough: the production losses, and the shortfall for other economic sectors were not considered, for example, as well as the lower market acceptance of products of vaccinated animals [29]. However, indicators were either considered separately (for each stakeholder) or recalibrated before to be considered jointly (in collective decisions). Therefore, assuming that in each region, the chosen indicators are proportional to the real impacts, the preceding limits of these indicators should not bias significantly the geographic variations of optimal strategies against FMD epizootics.

When the decision scope was national, vaccination-based strategies (both preventive and suppressive) showed the best epidemiological effectiveness as they allowed lowering the number of infected premises. Oppositely, this number was higher for strategies based on preemptive slaughter, the ability of which to control epizootics was weaker. This result may be explained by the fact that, when preemptive slaughter was applied, some infected premises were not identified: contact tracing could not be performed (as epidemiological inquiries were only performed in reported outbreaks), which indirectly favoured disease spread. In the particular case of selective preemptive slaughter, preemptive cull was targeted on animals with a low economic value, which were also the most abundant (small ruminants and non-breeding pigs): the strong decrease in the density of susceptible animals (only cattle and reproductive pigs remained) also induced a decrease of the number of diseased animals seen by farmers and veterinarians. The disease detection process was thus slowed in infected premises and the time to detection was lengthened, whereas disease spread was slowed but not stopped. These negative effects of preemptive slaughter were particularly marked in densely populated livestock areas (DPLA), where they often led to a saturation of available resources and to a loss of control over the epizootic course. The weight of DPLAs in the computation of national average impacts explained the predominance of vaccination in national-scope decisions. Because it allowed reducing the number of slaughtered herds, vaccination-based strategies were optimal for public opinion. For the same reason, vaccination allowed reducing the amount of compensations paid to farmers, and was also optimal for the government in national-scope decisions: the cost of vaccination was compensated by the decrease of the number of slaughtered herds (except for suppressive vaccination). The use of vaccine induced prolonged export bans, and was thus *a priori* not favourable to agro-food industries. However, such prolonged bans mainly affected exports to non-EU countries. As they represented <25% of total exports, vaccination-based strategies were also optimal for agro-food industries in national-scope decisions.

The average public costs and export losses were significantly reduced when the optimal decision was independently determined in each region, and the optimal strategies appeared much more variable than in national-scope decisions. Each of the 7 considered control strategies turned out to be optimal in at least one region, for a specific stakeholder (or for a specific combination of stakeholders' weights in a collective decision process). If vaccination-based strategies remained optimal in most regions for public opinion, the epizootics reduction of ban duration allowed preemptive slaughter-based strategies to be optimal in many regions for agro-food industries. Besides, from the government's point of view, vaccination and even preemptive slaughter induced significant extra-costs that were not always necessary to epizootic control: the basic stamping-out strategy was thus also optimal in many regions for the government.

The variations of stakeholders' weights in collective decisions induced various patterns, linked to the regional densities of cattle, swine and small ruminants. When they started in areas with high cattle and swine densities, epizootics were often severe and difficult to control. It was also the case when the density of small ruminants was high, whereas the density of cattle was medium or low. Because small ruminants show in general few clinical signs, infected premises were identified late, and dense populations of small ruminants allowed a prolonged disease spread, at a low level. In both types of areas (regions of group G1), vaccination-based strategies were optimal, except when the weight of agro-food industries was the strongest. In areas with a high or medium cattle density, infected premises were, on average, identified more quickly, even if the density of small ruminants was also high or medium. In these regions (regions of group G2), the stamping-out procedure became optimal when the government's weight was the strongest.

The variability of the optimal strategy between regions, and the connection between collective decision patterns and regional animal densities suggest that FMD control strategies could be usefully adapted to the local breeding conditions. To investigate the feasibility of such adaptive strategies, the proportion of impact variance attributable to the local conditions was estimated. If this proportion was high, adaptive strategies should be feasible: a carefully chosen set of variables describing the local conditions could be identified, that would allow determining the optimal strategy. In most cases, approximately 50% of variance impact was explained by the local conditions: simulated FMD epizootics thus showed a significant intrinsic variability and the predictive value of the initial conditions for determining the optimal strategy was not expected to be very high. A similar approach was used to investigate the feasibility of adaptive strategies that could be dynamically changed according to the evolution of the epizootic: for the public costs and for the number of slaughtered herds, knowing how the epizootic has evolved during the first weeks should allow predicting its total impact, and thus permit to adapt the control strategy.

In this study, it was assumed that control strategies were decided at the beginning of the epizootics and never changed afterwards. Such situations are not realistic: during real epizootics, decision-makers have to re-evaluate daily the chosen control strategy, and to decide whether this strategy has to be maintained or changed. Our results suggested that a steering tool could be designed for the control of FMD epizootics, based on the combination of a control panel and of a decision procedure based on control panel indicators.
